# Impact of sustainable lighting on guest psychological performance in coastal beach resorts

**DOI:** 10.1038/s41598-025-21024-3

**Published:** 2025-10-23

**Authors:** Aishah Wasti, Trupti Chauhan

**Affiliations:** Department of Design, Manipal School of Architecture and Planning, Manipal Academic of Higher Education, Manipal, Karnataka 576104 India

**Keywords:** Sustainable lighting, Resort typology, Psychological performance, Coastal region, Statistical analysis, Human behaviour, Psychology, Environmental social sciences

## Abstract

This research investigates the impact of sustainable lighting solutions on the psychological performance of guests in beach resorts, with a focus on the coastal region of Karnataka, India. Psychological performance is operationally defined as a combination of four measurable dimensions: mood enhancement, stress reduction, relaxation, and sleep quality, assessed using guest self-report surveys on a Likert scale. As the hospitality industry faces increasing pressure to implement sustainable practices without compromising guest comfort, this study explores whether and how lighting design contributes to guest well-being. A mixed-methods approach was adopted, combining quantitative surveys with qualitative insights. The study surveyed 100 participants across four beach resorts using structured questionnaires to examine guest preferences, satisfaction levels, and awareness of sustainable lighting practices. Findings reveal a strong guest preference for a balanced blend of natural and artificial lighting, with many recognizing the role of sustainable lighting in enhancing relaxation and reducing stress. Statistical analysis demonstrated a positive correlation between sustainable lighting features and improved guest psychological outcomes. The results offer valuable implications for resort designers and operators striving to align luxury hospitality with environmental responsibility. Integrating sustainable lighting not only enhances guest experience but also supports broader sustainability goals—ultimately promoting a more eco-conscious tourism model.

## Introduction

The hospitality industry, particularly the beach resort sector, faces increasing pressure to adopt sustainable practices while maintaining exceptional guest experiences^1^. Lighting design plays a pivotal role in achieving this balance, as it directly impacts both the ambiance and energy consumption of resort facilities. Sustainable lighting solutions, such as energy-efficient LED lighting and solar power, are increasingly recognized for their potential to reduce energy consumption while contributing to a resort’s environmental sustainability^2^. There is a limited understanding of the relationship between sustainable lighting and user psychology specifically within the context of beach resorts. While previous studies have explored the general effects of lighting on mood and well-being, there is a notable gap in understanding its psychological effects in resort environments^3^. Sustainable lighting is not merely about energy savings; it influences user comfort, mood, and relaxation, which are critical in a hospitality setting^4^. In beach resorts, where relaxation and recreation are key priorities for guests, understanding this relationship is even more essential^5^.

Psychological performance in this research has four discrete yet interconnected dimensions: mood (feeling good), relaxation (psychological relaxation), stress reduction (subjective ease of emotions), and sleep quality (facility in falling and maintaining sleep). They were assessed through guest feedback on specific questionnaire items rated on a 5-point Likert scale.

Beyond lighting specific research, this study was guided by experience and environment psychology to explain how lighting influences guest psychological performance. Experience Economy Theory posits that memorable experiences are staged through the four realms of entertainment, education, aesthetics, and escapism. Lighting plays a critical role in both the aesthetic and escapist realms by creating atmosphere and fostering immersion, which can also be used to facilitate relaxation and mood^6^. In a similar fashion, the Mehrabian–Russell model explains how environmental stimuli influence emotional responses of pleasure, arousal, and dominance, which then impacts behaviours of satisfaction and loyalty^7^. Within the Mehrabian–Russell model, sustainable lighting is a positive stimulus that elicits emotional responses of pleasure, arousal, and dominance that support guest relaxation, restorative rest, and well-being.

Identifying the optimal sustainable lighting solutions can lead to improved interior design practices in beach resorts, enhancing guest psychological comfort. As lighting is integral to interior ambiance, the study aims to provide practical recommendations for designers and architects to balance aesthetics, functionality, and sustainability in beach resort environments^8^. Studies have shown that properly designed lighting environments enhance the overall experience and emotional response of guests, especially in hospitality settings^9^. Sustainable lighting can reduce energy consumption by decreasing reliance on artificial lighting, leading to significant cost savings for resort owners. As resorts operate in energy-intensive environments, understanding how to optimize natural and artificial lighting will provide economic benefits while maintaining guest comfort (Thompson & Green, 2021). Research by^10^ indicates that energy-efficient lighting solutions, such as LEDs, can reduce energy costs by up to 40% in hospitality environments, contributing to the financial sustainability of resorts. By promoting sustainable lighting practices, the research by Sholanke, Fadesere and Elendu talks about contributing to broader sustainable design initiatives and help reduce the carbon footprint of beach resorts^2,11^. In a region like Coastal Karnataka, where ecological preservation is a priority, sustainable lighting solutions are essential to balance tourism and environmental sustainability^12^. As global environmental concerns rise, adopting such practices aligns with the sustainable tourism model that is becoming increasingly vital^13^. Understanding the psychological benefits of sustainable lighting is critical to creating healthier and more relaxing environments for beach resort guests. Sustainable lighting can enhance guest psychology by reducing stress and improving sleep quality, directly impacting their overall resort experience^14^. Research on lighting environments has shown that exposure to certain light temperatures and intensities can positively influence circadian rhythms and reduce mental fatigue^15^. The hospitality industry is increasingly adopting sustainability as a competitive advantage, and lighting forms an integral part of energy management systems^16^

This paper builds on Attention Restoration Theory, which suggests that environments with restorative qualities (e.g., being away, soft fascination, compatibility) can help reduce mental fatigue and enhance well-being. Lighting that mimics natural transitions (e.g., gradual changes in colour temperature and intensity) can provide these restorative qualities, even in indoor environments^17^. Additionally, the benefits of Circadian Lighting Theory are explored, which suggests being exposed to light that is in alignment with the body’s natural biological rhythm improves quality of sleep, mood, and cognitive functioning. Applying these theories to a resort environment offers a framework to understand how sustainable lighting design provides psychological performance benefits.

This research investigates the relationship between user psychology (mood enhancement, stress reduction, sleep quality and relaxation) within the context of sustainable lighting solutions in beach resort design.

### Research questions

i. How does sustainable lighting influence guest mood, relaxation, and sleep quality in coastal beach resorts?
ii. What is the relationship between guest preferences and the psychological effects of various sustainable lighting strategies?
iii. To what extent does awareness of sustainable lighting affect guest satisfaction?

### Objectives

i. To evaluate the psychological impact of sustainable lighting strategies on resort guests.
ii. To identify guest preferences for lighting in different resort spaces.
iii. To analyse the relationship between lighting comfort and well-being.
iv. To assess guest awareness of sustainable lighting and its influence on satisfaction levels.

### Literature review

The literature review for this research focuses on several key parameters that will be considered for statistical analysis regarding sustainable lighting solutions in beach resorts. The following parameters are essential for understanding the relationship between sustainable lighting design and user psychology:

### Artificial lighting

Artificial lighting plays a crucial role in creating comfortable and functional spaces within beach resorts. Studies show the importance of well-designed lighting in creating a welcoming atmosphere, highlighting the influence of lighting on perception and user experience^2^. Das and Paul investigated the factors influencing the use of artificial lights during the day in residential buildings, finding that home design and occupant behaviour play significant roles. Their research suggests that lighting design should consider the specific needs and preferences of residents^18^.

In the context of beach resorts, artificial lighting can be used to create a variety of atmospheres, from vibrant and energetic to calm and relaxing. By carefully selecting lighting fixtures, colours, and intensities, resorts can enhance the overall guest experience and create a memorable ambiance. Additionally, the use of smart lighting systems can allow for personalized lighting settings, catering to the individual preferences of guests^19^.

### Sustainable lighting

For the purpose of this study, “sustainable lighting” refers to lighting solutions that are energy-efficient, environmentally responsible, and psychologically beneficial. This includes the use of LED lighting, solar-powered fixtures, smart/dimmable systems, and lighting designs that support natural circadian rhythms. The definition emphasizes both the technological (source and controls) and experiential (comfort, ambience) aspects of lighting.

Sustainable lighting practices, such as LED lighting and smart lighting systems, offer significant benefits not only in terms of energy efficiency and environmental impact but also in enhancing the psychological well-being of guests^11^. According to El-Sayed and Abed, sustainable lighting in hotel lobbies contributes to a welcoming atmosphere that positively affects guests’ emotions and mental state. By using lighting systems that can be adjusted to create various atmospheres, resorts can influence mood, reduce stress, and promote relaxation, which are key factors in overall guest satisfaction^11^.

The ability to personalize lighting environments has a profound impact on guest well-being. Research by Heschong demonstrates how carefully designed lighting can influence mood, comfort, and overall satisfaction. Smart lighting systems, which allow for customization of light intensity and colour temperature, provide guests with greater control over their surroundings. This adaptability can create calming atmospheres in relaxation areas and more stimulating environments in communal spaces, enhancing the overall guest experience^20^.

Scientific literature links lighting temperature and intensity directly to physiological effects. Warm light temperatures (between 2700 K–3000 K) are associated with melatonin production and improved sleep onset, while cooler lights (5000 K–6500 K) are stimulating and suited for active areas^15^. Similarly, circadian lighting systems that mimic daylight cycles have been shown to stabilize mood and reduce anxiety, as highlighted in studies by^21^

While many studies claim warm-toned lighting is best for relaxation and sleep, other studies have shown that cooler white light, in fact, increases alertness, attentiveness and cognitive performance^22^. In this sense, lighting which has a cooling effect can be used more effectively when the activities require the occupation of the mind. The contradiction presented here is resolved by the context-based use of lighting: in leisure-oriented spaces, such as the hotel and beach resort, warm-toned lighting is preferred. However, in other spaces such as activities and multifunctional spaces, cooler lighting might be utilized. This approach of considering the use of light by context, would suggest that sustainable lighting practices within the resort do not follow a one colour temperature approach, but vary the tone and intensity by zone depending on its use.

Moreover, sustainable lighting practices like LED lighting are not only energy-efficient but also reduce the harshness of artificial lighting. Smith and Taylor discuss how softer, warmer lighting can help regulate circadian rhythms, improving sleep quality and reducing stress levels. The use of these lighting technologies allows resorts to create environments conducive to guest well-being, ultimately enhancing the overall experience^15^. Sustainable lighting, therefore, goes beyond its environmental benefits and plays a crucial role in psychological comfort and guest satisfaction.

### The role of lighting in resorts

Sustainable lighting is pivotal in enhancing guest experiences and promoting environmental responsibility within resort design.

A study by El-Sayed and Abed emphasizes the importance of integrating sustainability principles and advanced lighting technologies in hotel lobby areas to achieve energy efficiency and improve guest satisfaction^11^.

Incorporating natural light through architectural design can significantly enhance lighting performance and energy efficiency. Research indicates that the use of skylights and light wells can reduce energy consumption and CO₂ emissions in buildings, contributing to a more sustainable environment^23^.

In forest resorts, Bura and Narkhede analyse the need for lighting systems that balance safety and comfort with ecological preservation. Their study underscores the role of low-impact lighting systems, such as solar-powered LED fixtures, in minimizing disturbances to local wildlife while maintaining guest safety^24^.

Furthermore, the hospitality industry is increasingly adopting sustainable lighting solutions to create future-proofed interiors. Implementing LED lighting and smart control systems allows hotels to reduce their carbon footprint while maintaining aesthetic appeal and functionality^25^.

By focusing on sustainable lighting strategies, resorts can enhance guest satisfaction, align with global energy efficiency goals, and create memorable experiences that harmonize with their surroundings.

### Psychological impact of lighting on users

Understanding guest preferences is essential for designing lighting that meets their needs and expectations. Aditi and Roy explore the role of lighting in meditation resorts, highlighting the preference for natural light and warm tones^26^. This finding aligns with other studies that emphasize the importance of considering guest preferences when designing lighting, as it affects their psychological well-being and overall experience^21^.

Aampora’s research emphasize the importance of educating guests about sustainable practices and engaging them in environmentally friendly behaviours. In resorts, creating awareness about sustainable lighting options can foster a positive guest experience while reducing the environmental impact. Educational initiatives, such as signage explaining the benefits of energy-efficient lighting, are important for encouraging guests to engage in responsible practices^27^.

By considering guest preferences, resorts can create lighting environments that resonate with their clientele and enhance their overall satisfaction. For example, guests may prefer softer lighting in relaxation areas, while brighter lighting may be desired in areas where activities or socializing are common^24^. Additionally, understanding guest preferences regarding natural light can inform the design of spaces that maximize daylight and minimize the need for artificial lighting.

The literature review highlights several key gaps in existing research. First, there is limited knowledge about the specific psychological effects of lighting in beach resorts beyond general impacts on mood and well-being. Second, research is lacking on how to balance guest preferences with eco-friendly lighting solutions to create both sustainable and satisfying environments. Third, there is a need for more comprehensive studies on the ecological impact of resort lighting. Lastly, practical guidelines for optimizing lighting in different resort areas, such as lobbies and outdoor spaces, are scarce. Addressing these gaps will enhance understanding and support the development of sustainable, guest-centric lighting solutions.

By focusing on these parameters, the literature review aims to provide a comprehensive understanding of how sustainable lighting influences guest experiences and contributes to the overall sustainability of beach resorts. The findings will inform best practices and guidelines for resort operators seeking to enhance both guest satisfaction and environmental responsibility.

Beach resorts, in particular, are highly dependent on creating natural and aesthetically pleasing atmospheres for guests. As they often integrate outdoor and indoor spaces, lighting plays a significant role in enhancing the guest experience by blending artificial lighting with natural sunlight^28^. Additionally, beach resorts frequently cater to leisure and relaxation, where lighting impacts mood and well-being more profoundly compared to urban hotels or business-oriented accommodations^5^. Given these distinct factors, it is important to focus on beach resorts to understand how sustainable lighting can optimize guest well-being and resort functionality.

### Introduction of case studies

The scope of this research is focused on beach resorts located in Coastal Karnataka, India, a region known for its diverse coastal landscapes and growing tourism industry. Coastal Karnataka’s natural beauty, along with its commitment to eco-friendly and sustainable tourism practices, makes it an ideal setting for this research^29^. Furthermore, the region is home to a wide array of beach resorts that attract a global clientele, offering a relevant and diverse sample for studying the psychological impacts of lighting^30^. Ecologically sensitive coastal areas, like those in Coastal Karnataka, are also under significant environmental stress due to tourism development, making it crucial to investigate sustainable design solutions, including lighting, that can help mitigate this impact^29^. By focusing on this specific geographic region, the study aims to provide valuable insights for resort operators and designers seeking to enhance both guest satisfaction and sustainability in beach resorts.

**Fig. 1:**
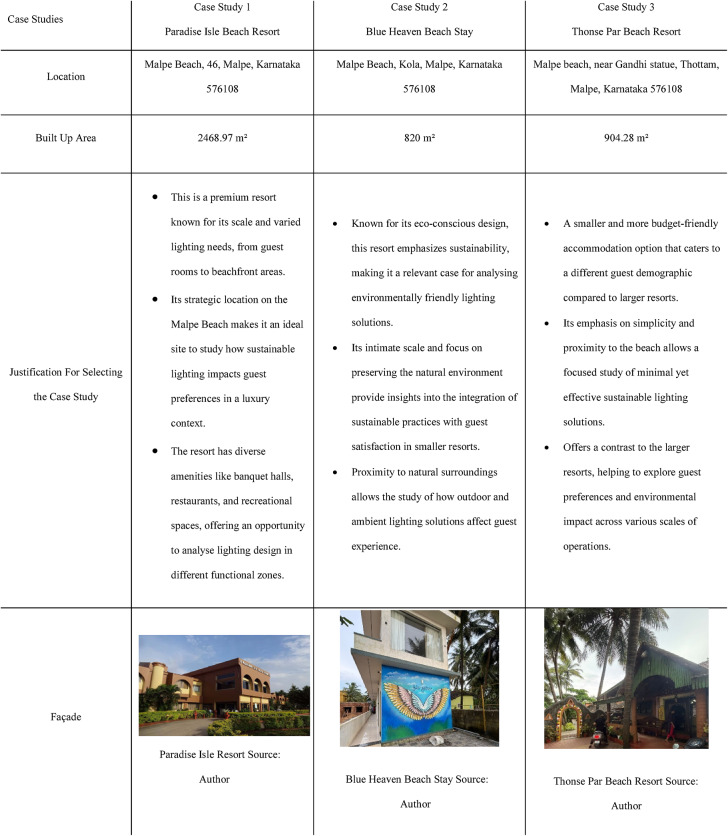
Brief introduction of case studies 1, 2 & 3.

### Case study 1: Paradise Isle Resort, Malpe, Karnataka

**Fig. 2:**
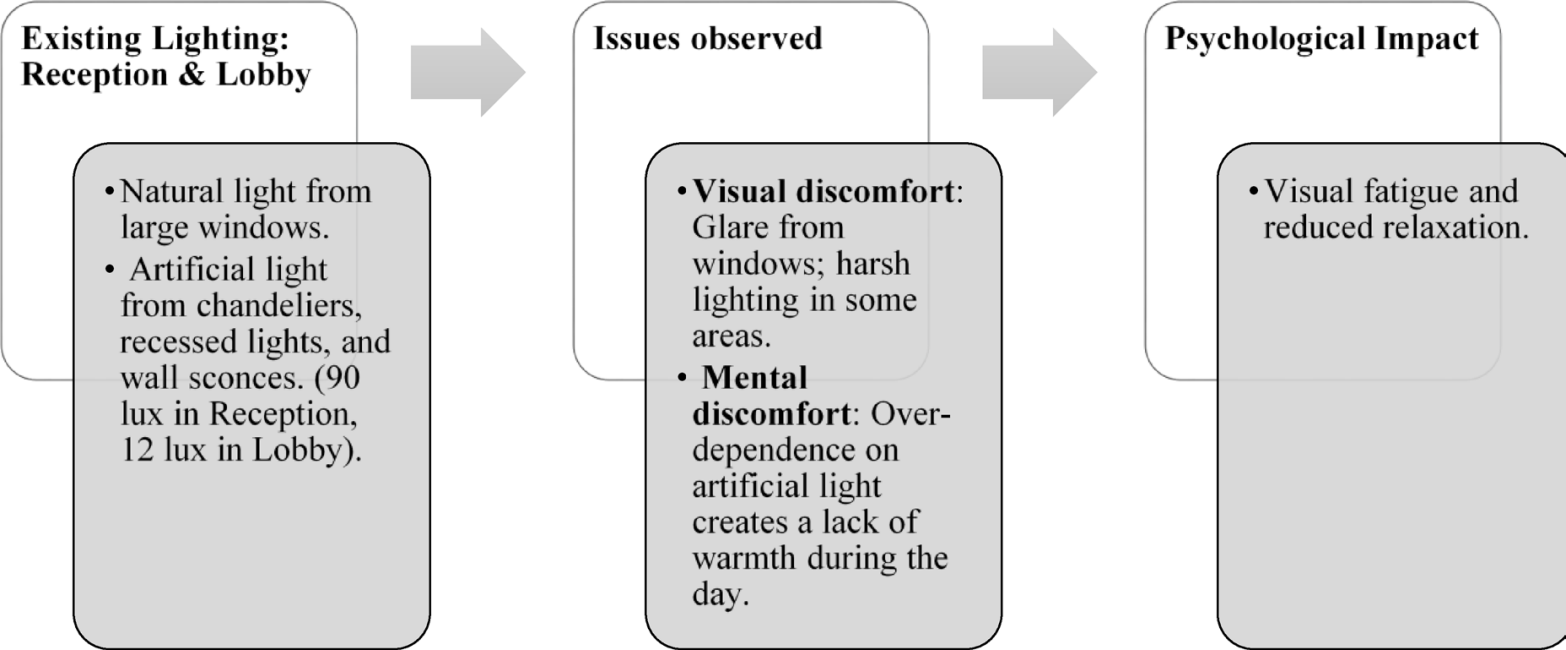
Reception & lobby lighting details of Paradise Isle Resort (Source: Author).

**Fig. 3:**
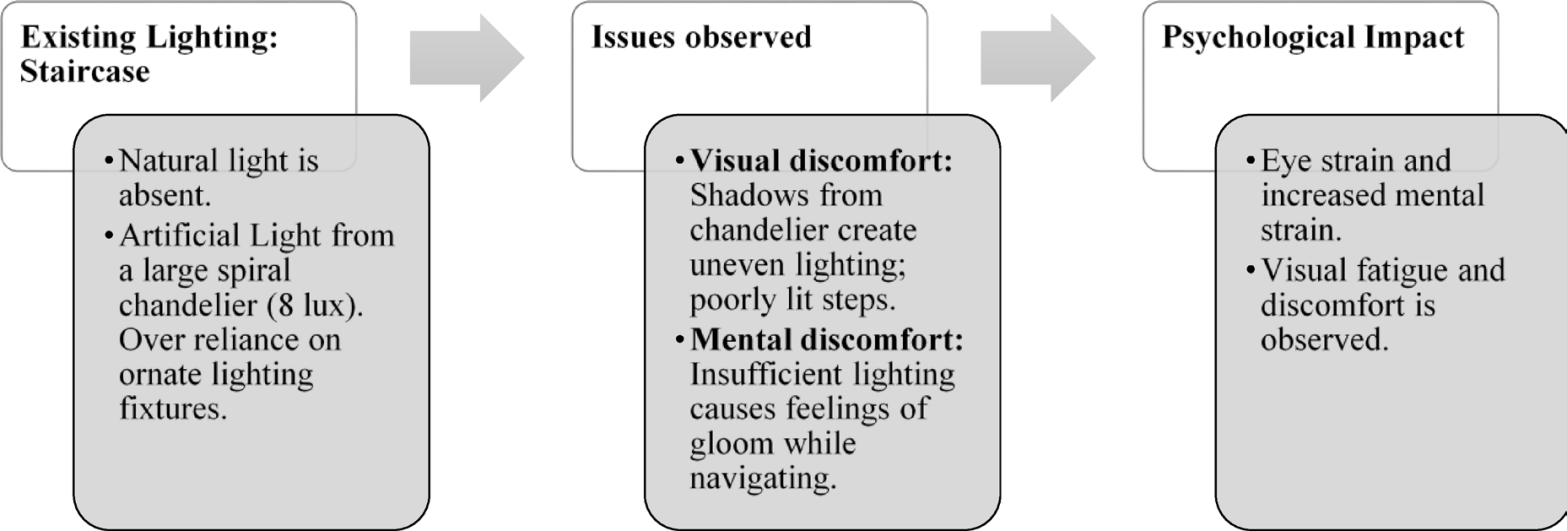
Staircase lighting details of Paradise Isle Resort (Source: Author).

**Fig. 4:**
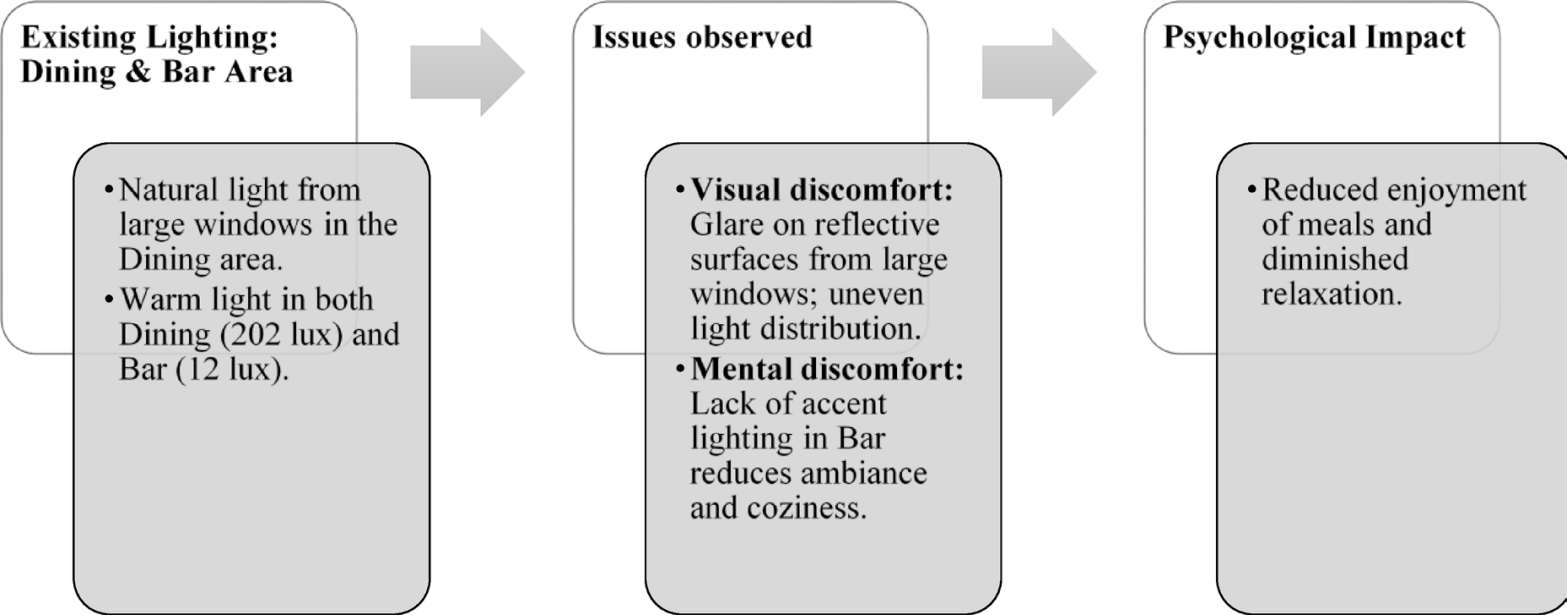
Dining & bar area lighting details of Paradise Isle Resort (Source: Author).

**Fig. 5:**
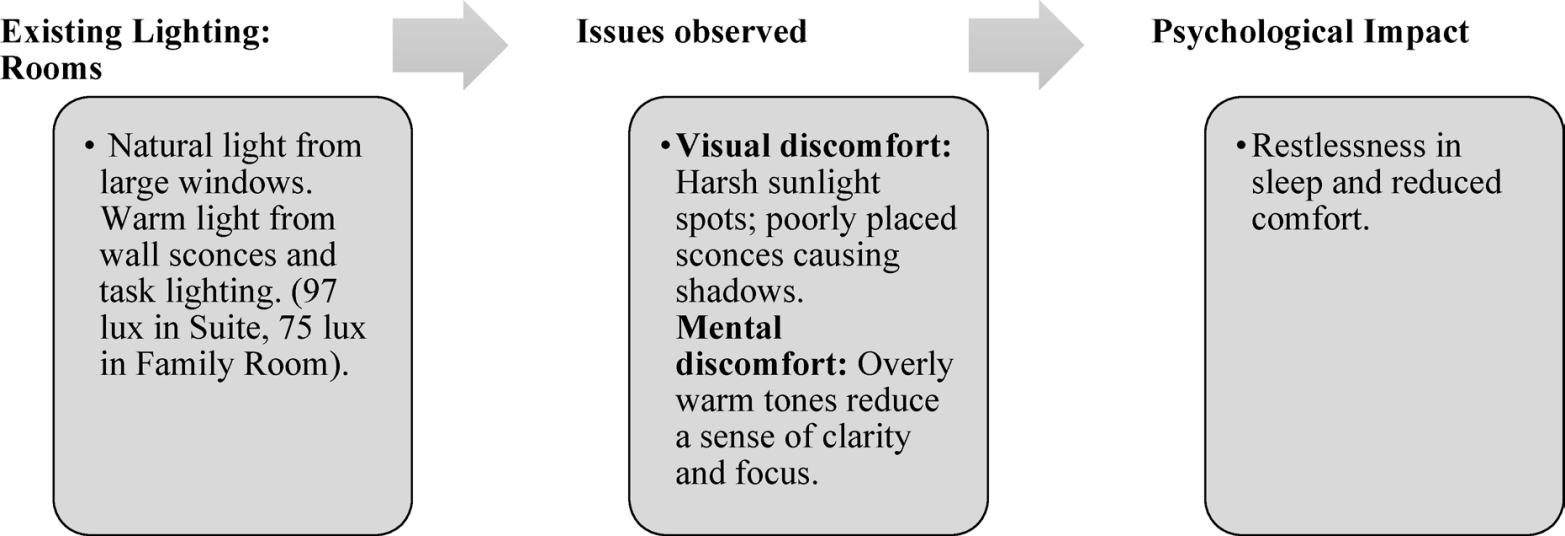
Rooms Lighting details of Paradise Isle Resort (Source: Author).

**Fig. 6:**
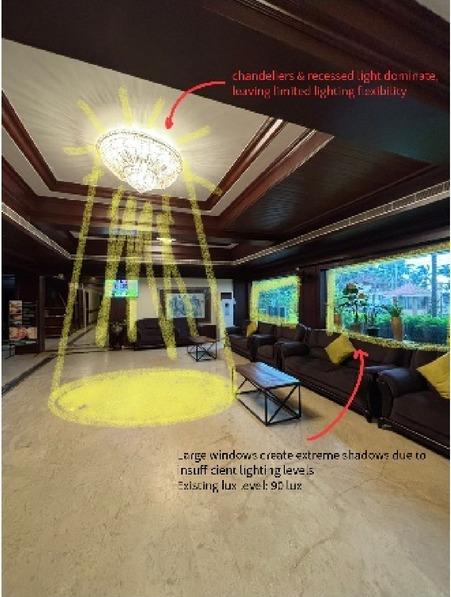
Reception area (Source: Author).

**Fig. 7:**
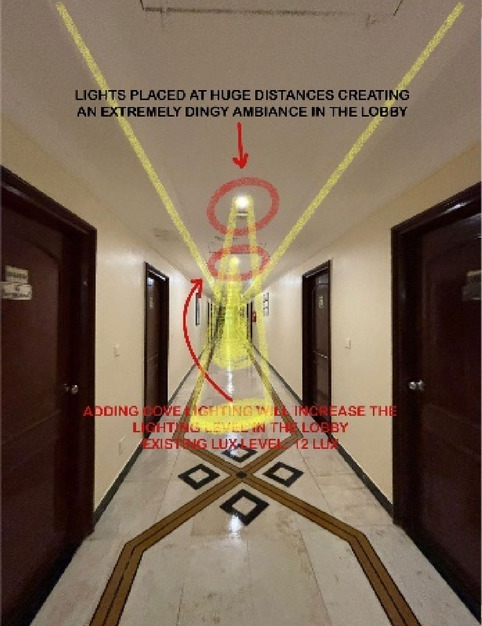
Lobby (Source: Author).

**Fig. 8:**
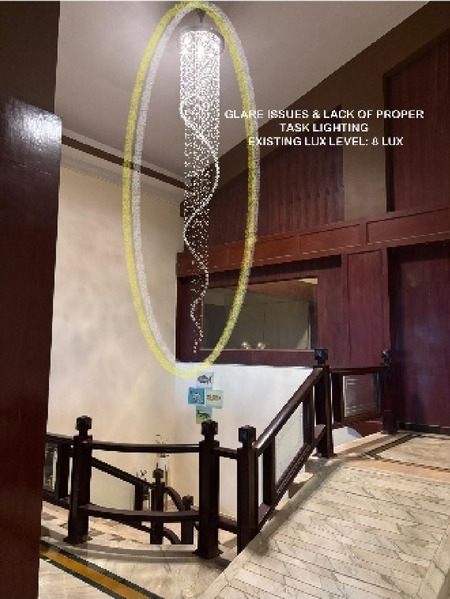
Staircase (Source: Author).

**Fig. 9:**
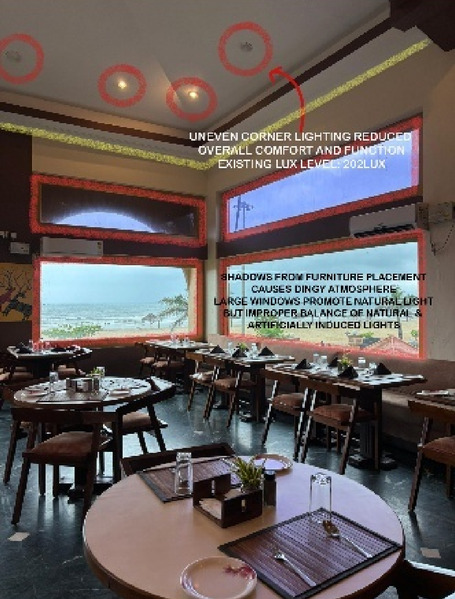
Dining (Source: Author).

**Fig. 10:**
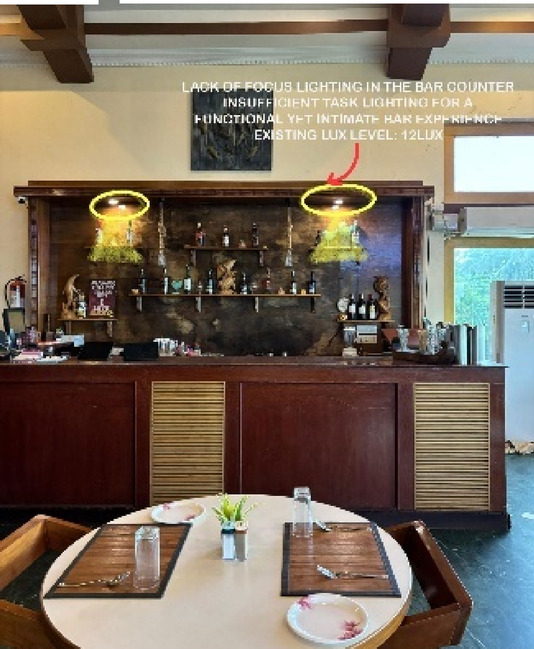
Bar area (Source: Author).

Paradise Isle Beach Resort as seen in Fig. 1 is a well-established beachfront destination known for its hospitality and scenic ocean views. The resort offers a blend of traditional and contemporary design elements to enhance guest comfort and relaxation. This case study highlights the critical impact of balanced lighting design on visual comfort, relaxation, mood and user experience across various functional spaces as explained in Figs. 2, 3, 4 and 5. Figures 6, 7, 8, 9 and 10 give a brief understanding of the technical and visual aspects of lighting in the different spaces of the resort and how the functionality of the lights used is portrayed in those spaces.

### Case Study 2: Blue Heaven Beach Stay, Malpe, Karnataka

**Fig. 11:**
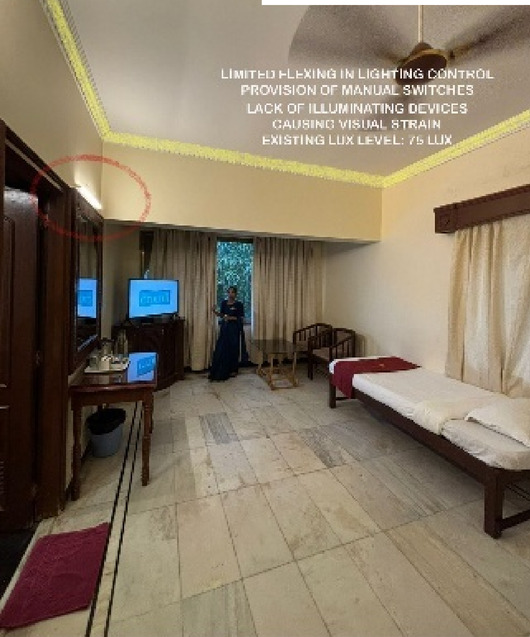
Family room (Source: Author).

**Fig. 12:**
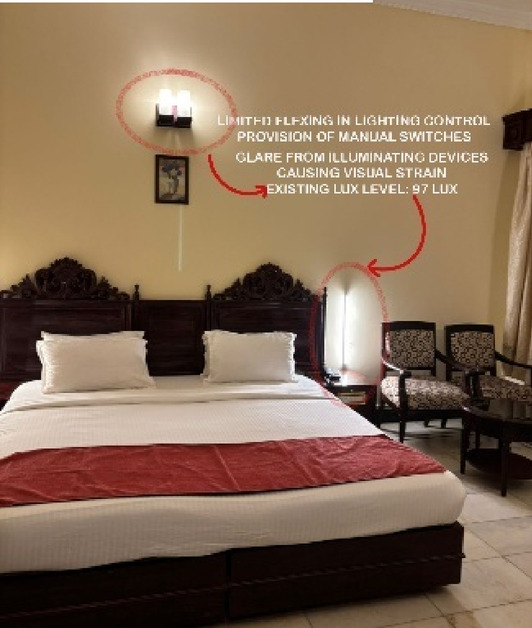
Suite room (Source: Author).

**Fig. 13:**
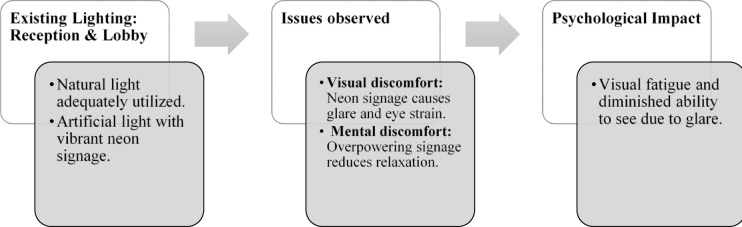
Reception & lobby lighting details of Blue Heaven Beach Stay (Source: Author).

**Fig. 14:**
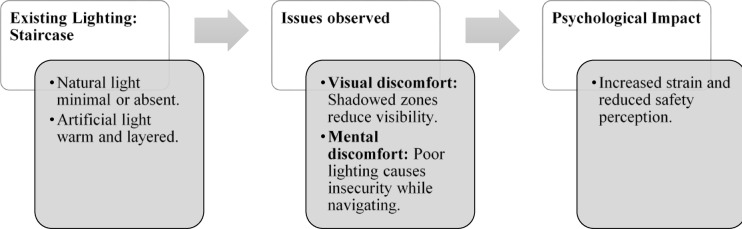
Staircase lighting details of Blue Heaven Beach Stay (Source: Author).

**Fig. 15:**
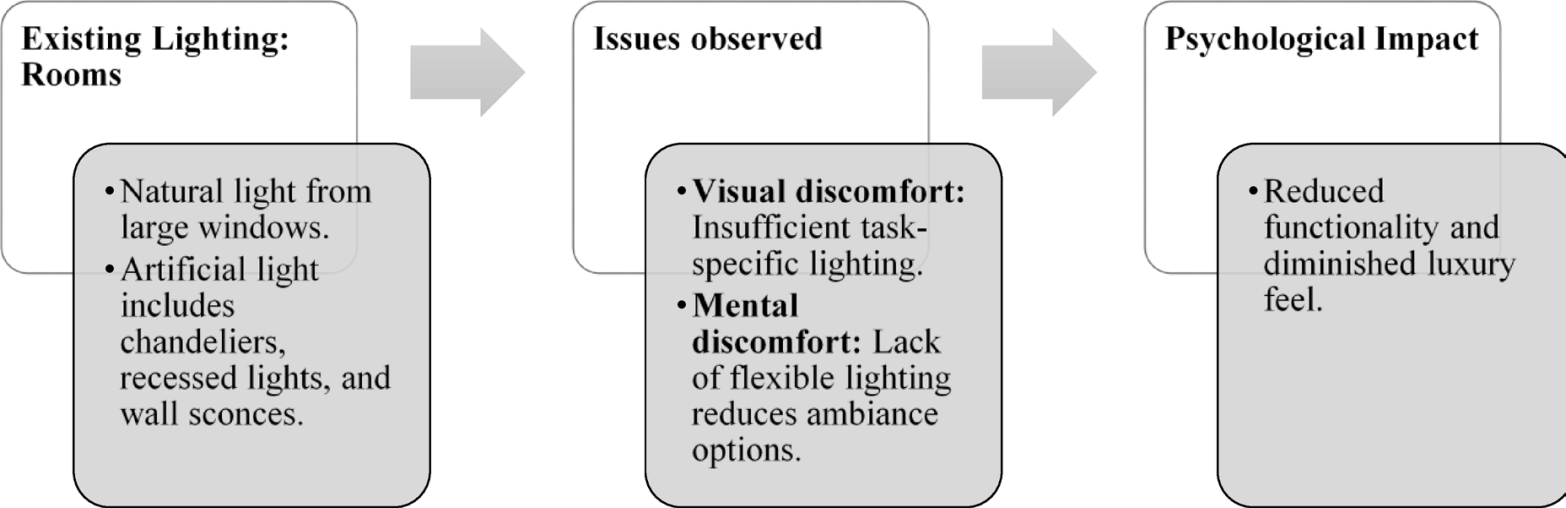
Staircase lighting details of Blue Heaven Beach Stay (Source: Author).

**Fig. 16:**
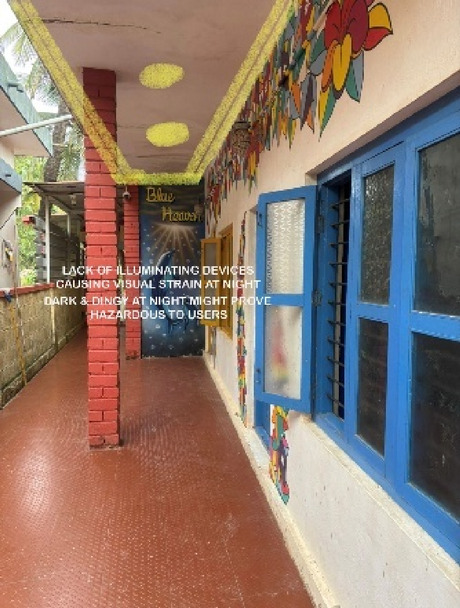
Lobby (Source: Author).

**Fig. 17:**
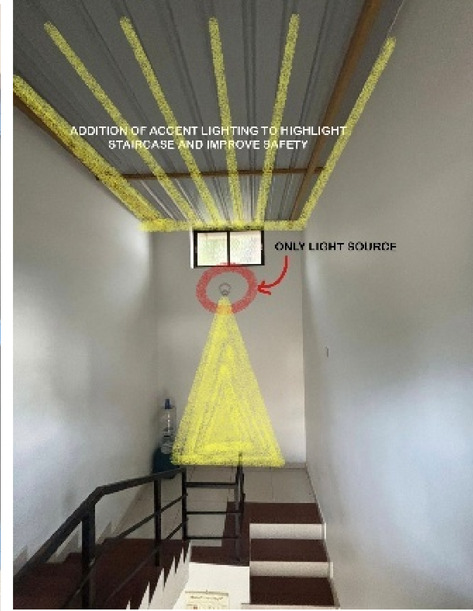
Staircase (Source: Author).

**Fig. 18:**
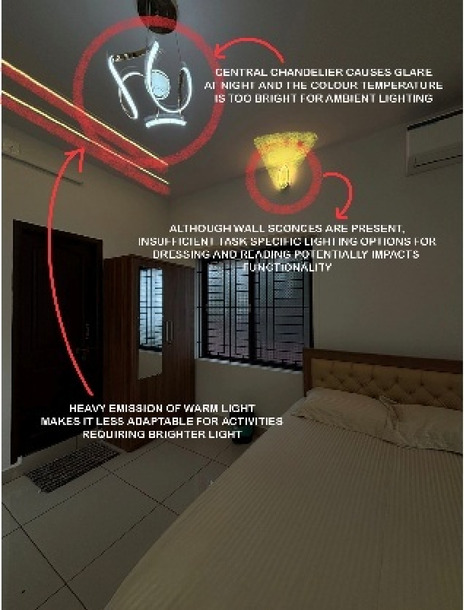
Suite room (Source: Author).

**Fig. 19:**
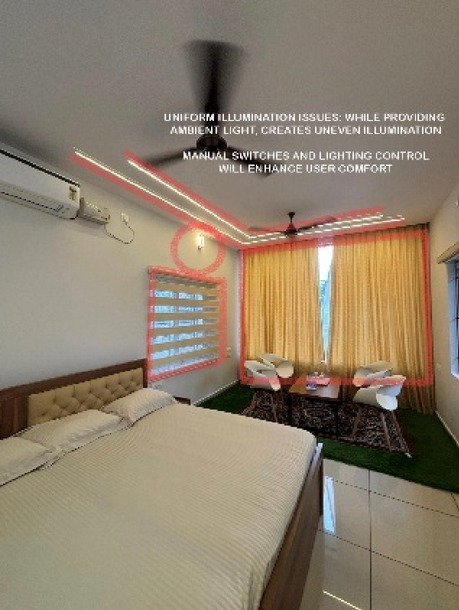
Suite room (Source: Author).

Blue Heaven Beach Stay as seen in Fig. 1 is a boutique accommodation that focuses on simplicity and tranquillity. With its intimate setting and minimalistic design, it provides guests with a peaceful retreat close to the shoreline (Figs. 11 and 12). The different issues observed and the psychological impact these issues causes have been discussed in Figs. 13, 14 and 15. The visual impact the lighting creates has been portrayed in the Fig. 16, 17, 18 and 19. The overlapped sketches demonstrate the usage of lighting in various areas of the beach stay.

### Case Study 3: Thonse Par Beach Resort, Malpe, Karnataka

Thonse Par Beach Resort as seen in Fig. 1 is designed to offer a nature-integrated experience with sustainable practices. Surrounded by coastal beauty, the resort aims to provide a serene and eco-friendly environment for guests.

Figures 20, 21, 22 and 23 demonstrates the lighting conditions of the spaces and the visual and psychological impact it has on the users. The demonstrative sketches can be observed in the Figs. 24, 25, 26, 27 and 28.

**Fig. 20:**
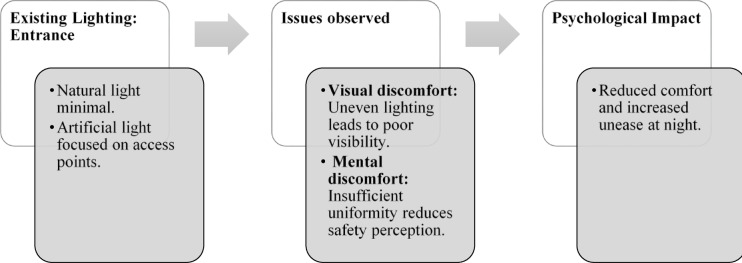
Entrance lighting details of Thonse Par Beach Resort (Source: Author).

**Fig. 21:**
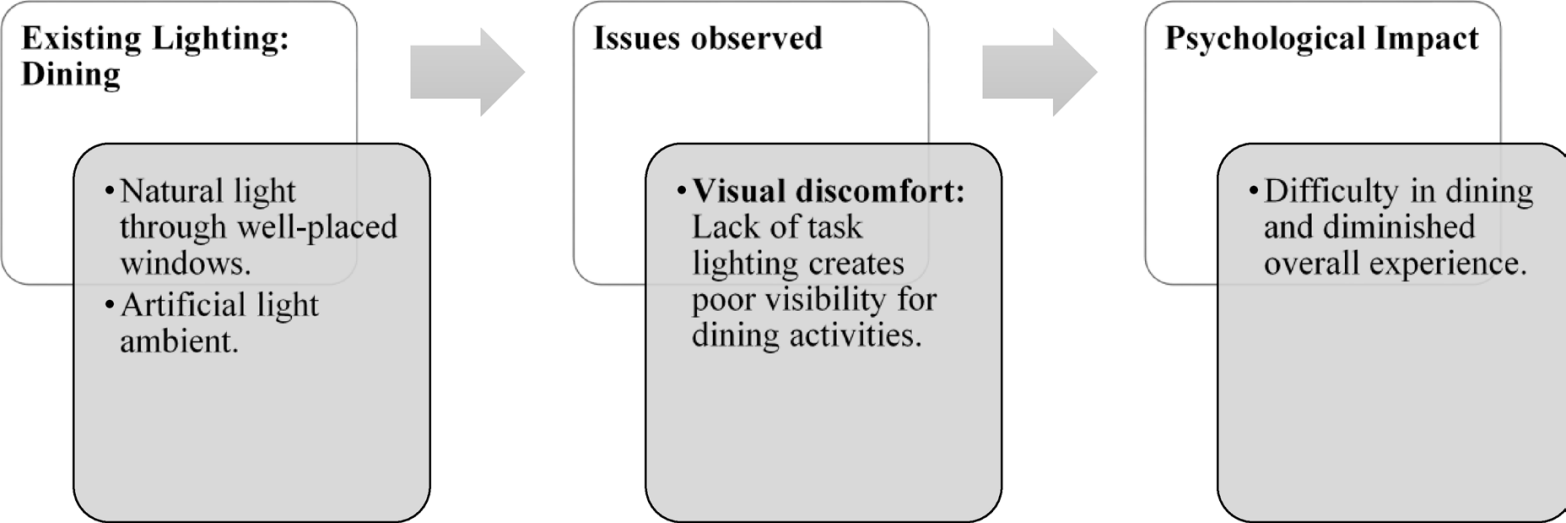
Dining LIGHTING DETAILS of Thonse Par Beach Resort (Source: Author).

**Fig. 22:**
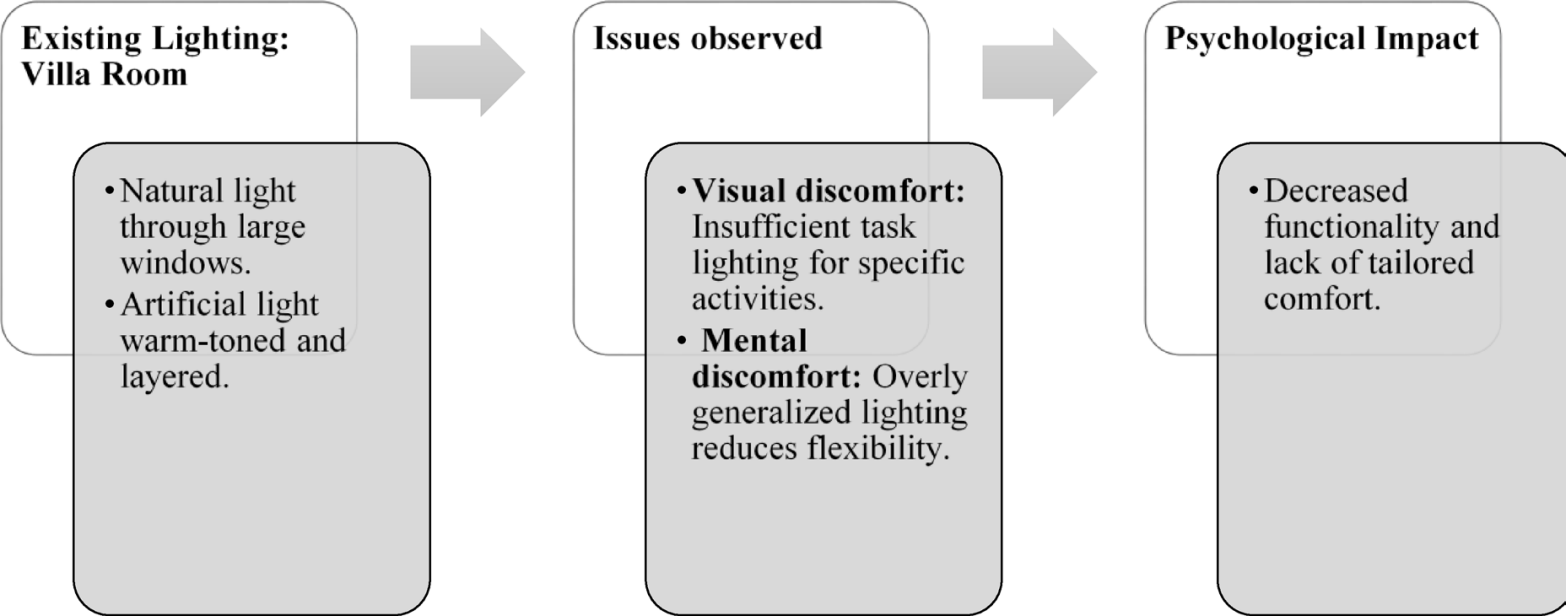
Villa room lighting details of Thonse Par Beach Resort (Source: Author).

**Fig. 23:**
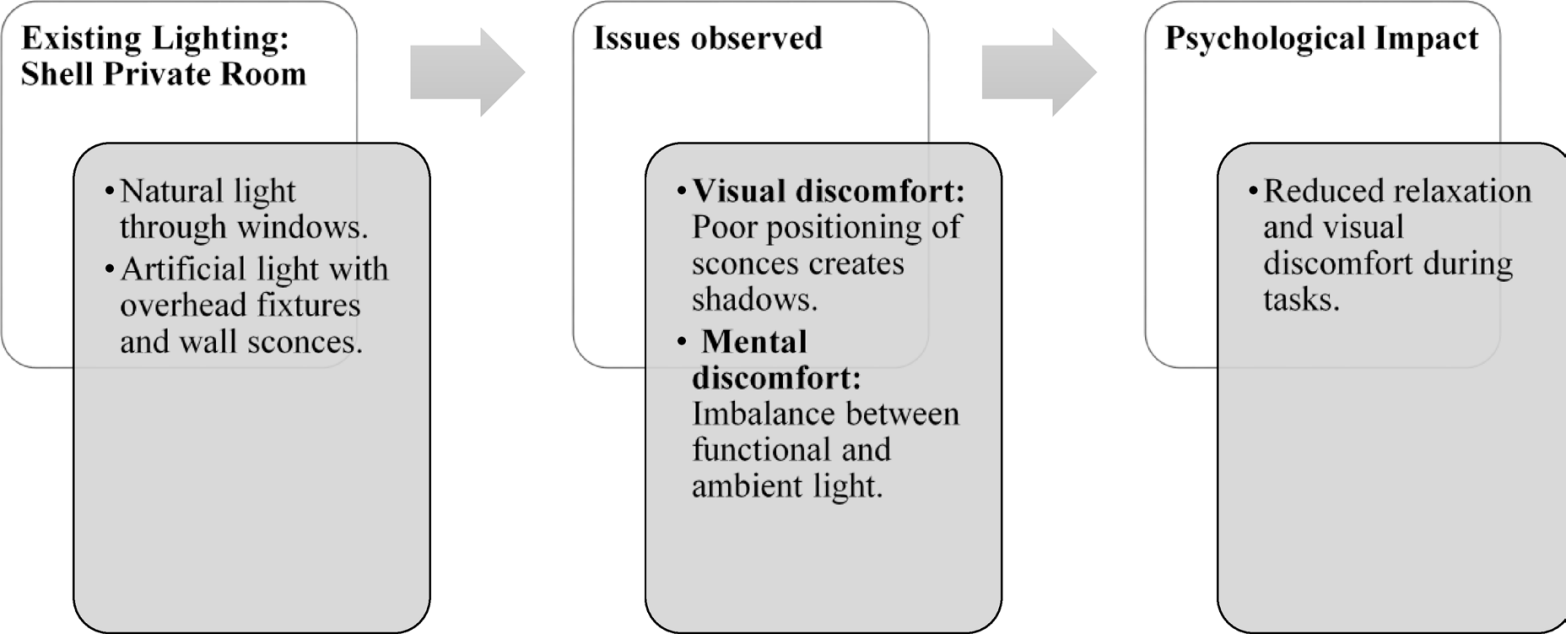
Shell private room lighting details of Thonse Par Beach Resort (Source: Author).

**Fig. 24:**
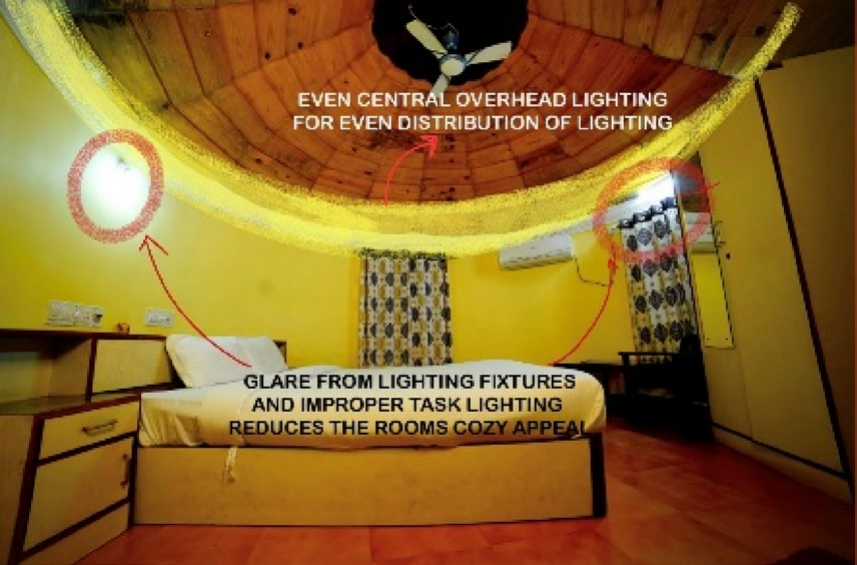
Family villa room (Source: Author).

**Fig. 25:**
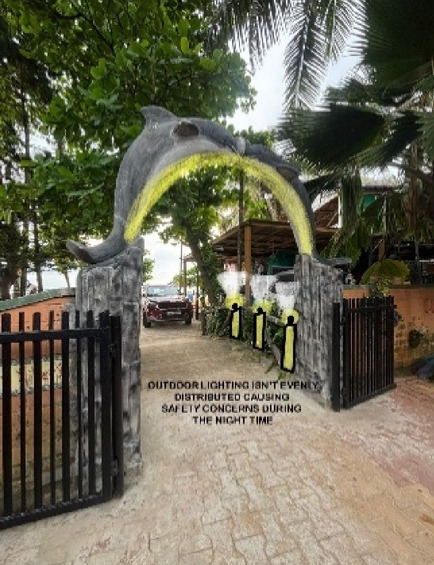
Entrance (Source: Author).

**Fig. 26:**
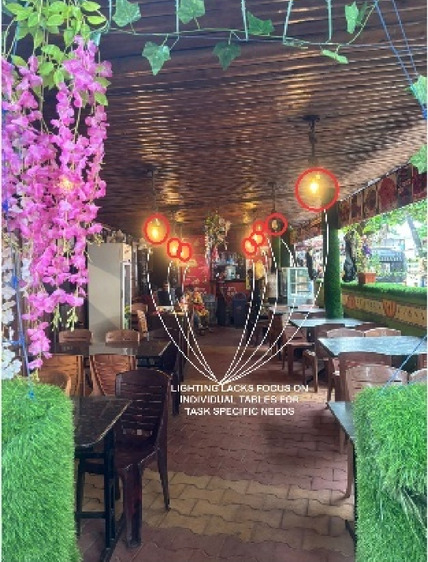
Dining (Source: Author).

**Fig. 27:**
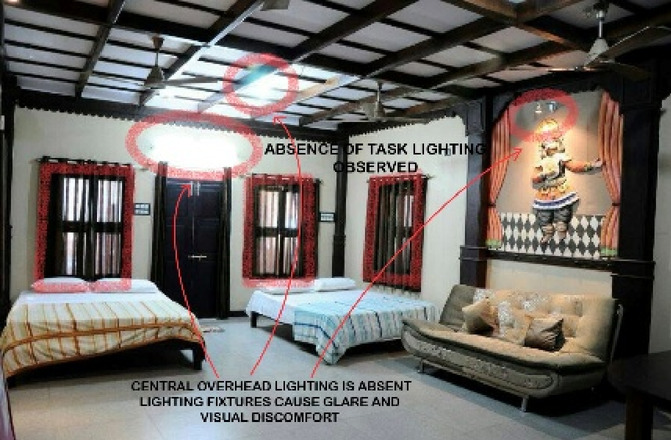
Non-AC villa room (Source: Author).

**Fig. 28:**
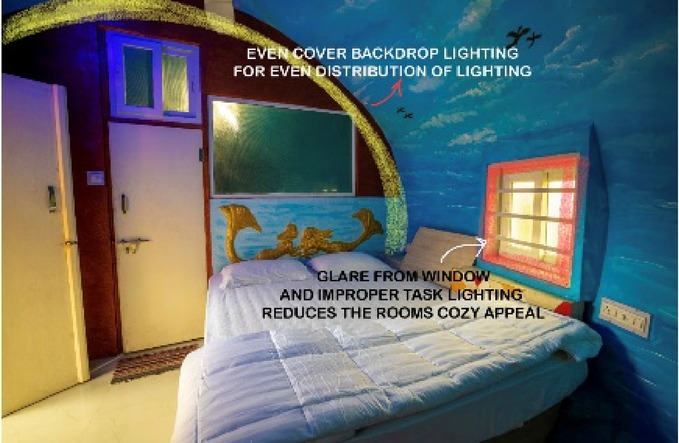
Shell private room (Source: Author).

## Research methodology

A qualitative and quantitative methodology will be utilized to gather comprehensive data on the psychological effects of lighting on guests. Quantitative data will be collected through questionnaires distributed to resort guests/users, focusing on how sustainable lighting impacts their psychology. This will be complemented by qualitative insights to provide deeper context. Statistical analysis will then be applied to identify correlations between sustainable lighting practices, guest satisfaction, experiences, and its correlation.

**Fig. 29:**
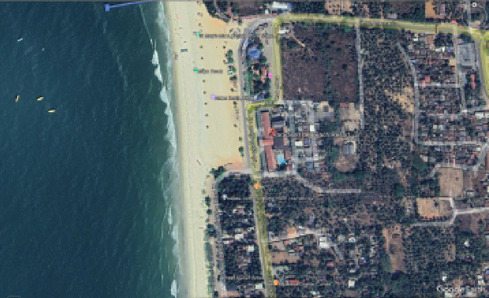
Paradise Isle Resort Location. Source: Google Earth Pro. Location: Paradise Isle Beach Resort, 46, Malpe, Karnataka 576,108 (Figs. 29 and 32). Site Area: 2468.97 m^2^.

**Fig. 30:**
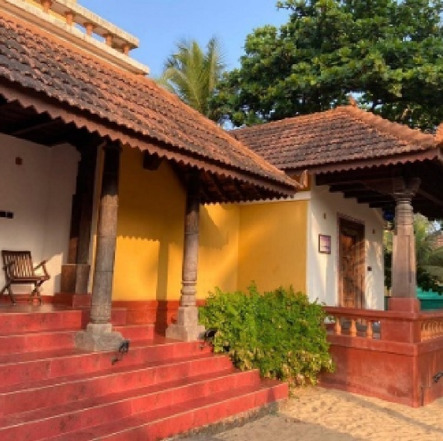
Sai Radha Beach Resort Source: www.tripadvisor.com. Location: Sai Radha Beach Resort, Bikriguthu Road, Mulluru village, Uchilla, Padu, Karnataka 574,117 (Figs. 30 and 31). Site Area: 1587.65 m^2^.

**Fig. 31:**
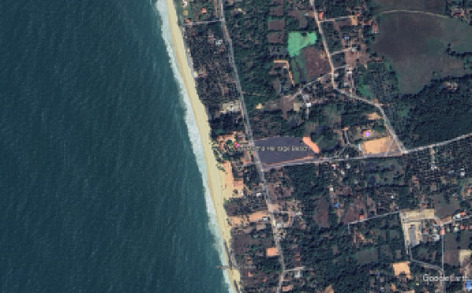
Sai Radha Beach Resort Location. Source: Google Earth Pro. Location: Sai Radha Beach Resort, Bikriguthu Road, Mulluru village, Uchilla, Padu, Karnataka 574,117 (Figs. 30 and 31). Site Area: 1587.65 m^2^.

**Fig. 32:**
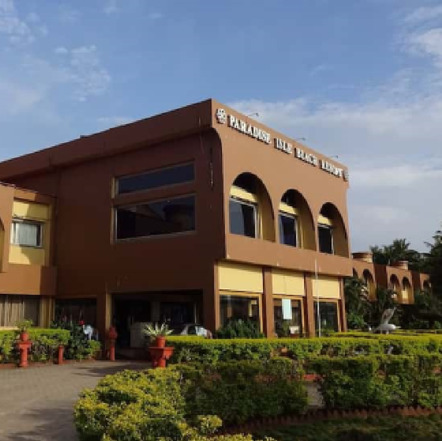
Paradise Isle Resort Source: yatra.com. Location: Paradise Isle Beach Resort, 46, Malpe, Karnataka 576,108 (Figs. 29 and 32). Site Area: 2468.97 m^2^.

However, there are some limitations to this research. The study’s focus on a single geographic region, Coastal Karnataka, may limit the generalizability of the findings to beach resorts in other regions. Additionally, while the use of quantitative methods allows for comprehensive statistical analysis, it may not capture the full range of subjective experiences and attitudes that qualitative research could reveal. By understanding the psychological effects of lighting and the potential benefits of sustainable practices, resort operators can make informed decisions to enhance both guest satisfaction and environmental sustainability in their facilities.

The conceptual model of this research is based on the relationship between sustainable lighting variables (e.g., energy-efficient lights, warm tone lighting, dimmable controls, natural light integration) and guest psychological outcomes (mood enhancement, relaxation, sleep quality, satisfaction). The model hypothesizes that sustainable lighting positively influences guest psychological performance and overall satisfaction.

All questionnaire items related to sustainable lighting were measured using a 5-point Likert scale (1 = Strongly Disagree to 5 = Strongly Agree). This scale was applied consistently across both the pilot and main study to evaluate guest perceptions, preferences, and psychological responses to lighting environments.

### Pilot study

The pilot study involved collecting preliminary data from 20 participants across two resorts: Paradise Isle Resort in Malpe (Figs. 29 and 32) and Sai Radha Heritage Resort (Figs. 30 and 31). The primary objective of this phase was to refine the methodology used, ensure its reliability and validity, and evaluate the feasibility of the research approach. This step also facilitated the identification of potential challenges and the implementation of necessary adjustments to enhance the effectiveness of the main study.

In addition to the structured survey, qualitative data was collected through open-ended questions at the end of the questionnaire. These responses were thematically analyzed to extract patterns related to guest perceptions of lighting comfort and ambiance. Thematic coding was performed manually to identify key psychological outcomes such as relaxation cues and lighting preferences in specific resort zones. Quotes and insights were integrated into the findings section where relevant.

### Questionnaire structure

**Fig. 33:**
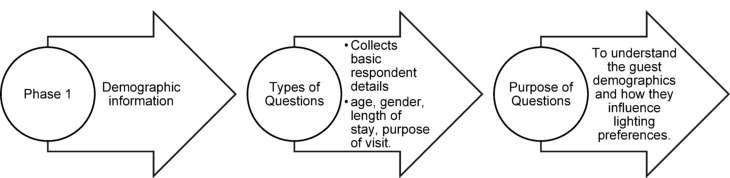
Phase 1 Questionnaire structure of pilot study (Source: Author).

**Fig. 34:**
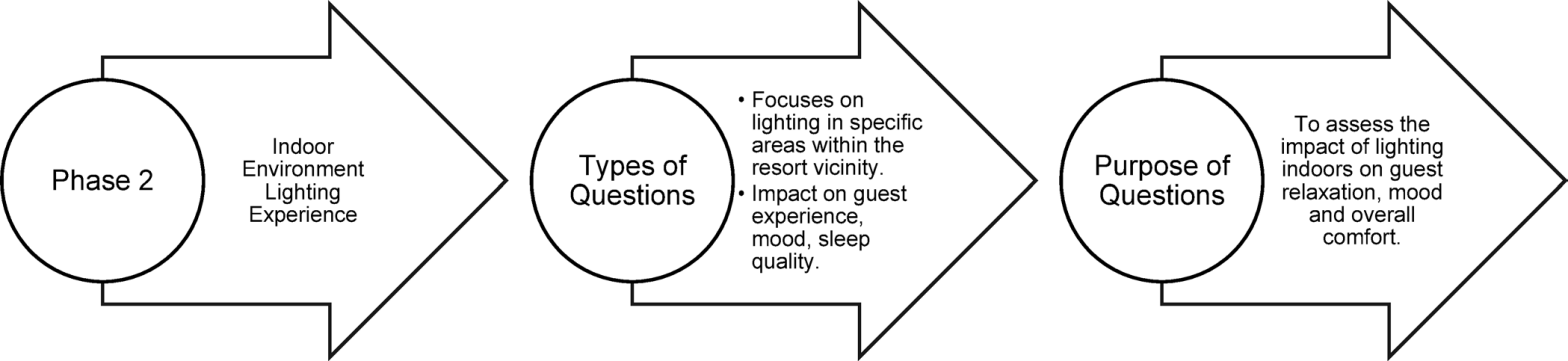
Phase 2 Questionnaire structure of pilot study (Source: Author).

**Fig. 35:**
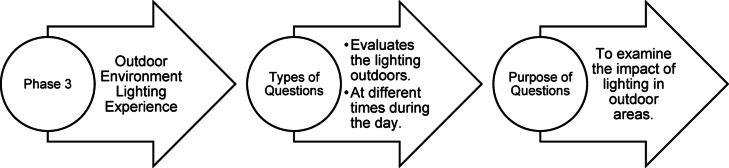
Phase 3 Questionnaire structure of pilot study (Source: Author).

**Fig. 36:**
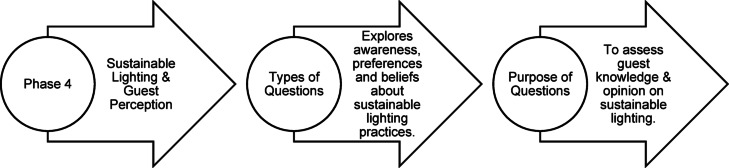
Phase 4 Questionnaire structure of pilot study (Source: Author).

The questionnaire was divided into four phases as shown in Figs. 33, 34, 35 and 36:

In Phase 4 of the survey, guests were presented with various sustainable lighting strategies through visual comparisons to understand and make informed choices about lighting options. The main areas explored were:

i. *Energy-Efficient Lighting* Guests compared energy-efficient LED lighting with traditional incandescent lighting to assess which option contributed more to a relaxed and comfortable environment.
ii. *Dimmable and Adjustable Lighting* Guests evaluated the importance of dimmable lighting controls for their relaxation and comfort, examining how adjustable lighting affects their environment.
iii. *Warm-Toned Lighting for Sleep Quality* Guests were asked to compare warm-toned lighting at night with cooler-toned lighting to determine its potential impact on sleep quality.
iv. *Smart Lighting Systems* Guests were introduced to the idea of smart lighting systems that adjust automatically based on the time of day and assessed their interest in such systems for improving mood and relaxation.
v. *Light Pollution Reduction* Guests compared scenarios of harsh versus soft, steady lighting to understand how light pollution reduction affects sleep quality and overall comfort.
vi. *Task-Specific Lighting* Guests were asked to evaluate task-specific lighting preferences, such as focused lighting for reading or soft ambient lighting for relaxation, to understand its effect on comfort and mood.
vii. *Mood-Enhancing Lighting Design* Guests were asked about their reactions to lighting that mimic natural light cycles throughout the day, considering its impact on mood and well-being.
viii. *Sustainable Lighting Awareness* Guests evaluated their perception of using sustainable and energy-efficient lighting and its influence on their overall experience and awareness of the resort’s environmental practices.

The goal was to help guests understand the connection between sustainable lighting choices and their psychological comfort, mood, and sleep quality, while also promoting awareness of the environmental benefits of sustainable lighting practices.

The internal consistency of the Likert-scale items was assessed using Cronbach’s Alpha. The results showed α = 0.82 for mood enhancement, α = 0.80 for relaxation, α = 0.78 for stress reduction, and α = 0.81 for sleep quality, all exceeding the recommended 0.70 threshold for good reliability. Items based on preference ranking or single comparative choices were excluded from this calculation, as reliability analysis is not applicable to those formats.

### Observations in pilot study

*Phase 1* Students represent the largest segment of respondents, with most staying for 1–2 nights, indicating a trend towards short trips.

*Phase 2* A combination of natural and artificial lighting is preferred, suggesting guests appreciate a balanced approach. Most express general satisfaction with indoor lighting, particularly in lobby areas.

*Phase 3* Guests show a preference for outdoor lighting during the day, though satisfaction levels are high for both day and night settings.

*Phase 4* Lighting is seen as moderately effective in reducing stress and promoting relaxation, with a notable impact on sleep quality.

*Phase 5* A moderate level of awareness about sustainable lighting practices was noted, with a significant majority perceiving its importance and benefits.

The pilot study provided valuable insights into guest perceptions of sustainable lighting, confirming its impact on psychological performance in coastal beach resorts. The findings indicated consistent patterns and validated the research framework, justifying the progression to the main study for a more comprehensive analysis. Overall, the pilot findings provide a solid basis for further exploration of sustainable lighting’s impact on guest psychology & wellbeing.

### Data analysis of main study

Sample Size & Locations: The study was conducted with a sample size of 100 respondents across four beach resorts in Coastal Karnataka.

The survey used a convenience sampling technique and asked 100 respondents from four beach resorts along Karnataka’s coastline to represent the diverse range of accommodation options from affordable stays to luxury resorts. The method intended to provide an authentic cross-sample of beach resort visitors. Among the respondents were 56% male and 44% female participants, consisting of 72% domestic visitors and 28% foreign guests aged between 18 and 55 years. The sample size was set based on practical convenience and statistical workability requirements to ensure sufficient variability among resort categories. The choice was also guided by the results of a pilot study using 20 subjects, which confirmed the structure of the questionnaire and its reliability for large-scale data collection.

i. Paradise Isle Beach Resort: 46, Malpe, Karnataka 576,108
ii. Sai Radha Heritage Beach Resort: Bikriguthu Road, Mulluru village, Uchilla, Padu, Karnataka 574,117
iii. Blue Heaven Beach Stay: Near Malpe, Kola, Malpe, Karnataka 576,108
iv. Thonse Par Beach Resort: 9M6X + W7F, beach, near Gandhi statue, Thottam, Malpe, Karnataka 576,108

This section examines the diverse lighting experiences of guests in resort, with a focus on sustainable lighting options and their impact on guest comfort, mood, and relaxation. The research intends to identify the role played by lighting in enhancing the relaxation, sleep quality, and overall satisfaction of guests by analysing the preferences of resort guests regarding both natural and artificial lighting.

These findings underscore the importance of a balanced approach to lighting that caters to both relaxation and activity-based needs, ultimately enhancing the overall guest experience.

### Statistical analysis

Prior to running regression analyses, assumptions of normality, linearity, and homoscedasticity were tested and met. This ensured that the data fulfilled the necessary conditions for valid parametric statistical testing.

The statistical analysis section highlights the integration of mixed methods to provide a comprehensive understanding of guest psychology with sustainable lighting in resorts. By using this methodology, the research not only identifies statistical patterns but also uncovers the reasons behind guest preferences, offering a balanced and in-depth analysis of the relationship between lighting design and user psychology.

Proposed Recommendations for guest satisfactions level against lighting levels to evaluate the statistical analysis are given in Table 1:

**Table 1:** Proposed recommendations for guest satisfactions level.

Standard deviation	0.5	0.6–0.7	0.8	0.9
Guest mood satisfaction level	Poor	Average	Moderate	Strong

Table 1 presents proposed recommendations for guest satisfaction levels based on the standard deviation of responses, not on the mean score directly. A lower standard deviation indicates greater agreement among respondents (e.g., SD = 0.5, “Poor” level suggests uniform dissatisfaction), while higher values indicate a broader range of opinions. This classification is distinct from the conventional interpretation of mean Likert scale scores.

The means and corresponding standard deviations of indoor lighting (a combination of natural and artificial lighting) experiences are given in Table 2. The results indicate that there is a mixed perception of lighting in the bar, dining areas, lobbies, lounge areas, and rooms of the resorts, with satisfaction levels ranging from poor to average across different spaces.

i. *Bar and Dinning* The lighting in the bar and dining area has a mean score of 3.42 ± 0.607 indicating that guests find it of average satisfaction.
ii. *Dining area* Likewise, the lighting (Combination of natural & artificial lighting) for dining has a mean score of 3.84 ± 0.509 indicating that guests find it of poor satisfaction for dining purposes.
iii. *Bar area* On the other hand, the contribution of lighting (Combination of natural & artificial lighting) in the bar area has a mean score of 3.75 ± 0.596 indicating that guests find the bar area lighting of poor satisfaction.
iv. *Lobby area* Lastly, the artificial lighting in the lobby area has a mean score of 3.8 ± 0.795 which indicates average satisfaction in the lobby.
v. *Lounge area* On the other hand, artificial lighting in the lounge area showed a slightly higher mean of 3.51 ± 0.701 indicating that the guests were averagely satisfied with the ambiance it created.
vi. *Rooms* In the same way, the impact of lighting i.e. combination of natural and artificial lighting within rooms were observed to help guests relax, indicated by a mean of 3.97 ± 0.66, that reflected a generally poor satisfaction of lighting’s role in promoting relaxation.

**Table 2:** Resort lighting experience.

Indoor lighting experience (Mood)	Mean	Standard deviation
Comfort of the natural & artificial lighting in the bar and dining	3.42	0.607
Sufficiency of the current lighting for dining	3.84	0.509
Contribution of lighting to the desired atmosphere in the bar area	3.75	0.596
Comfort of lighting in the lobby area	3.80	0.795
Comfortable of Artificial lighting in the lounge area for evening	3.51	0.701
Lighting in your room to help for relaxation	3.97	0.662
Lighting’s impact on sleep quality	3.94	0.767
Natural lighting in room during the day time	3.79	0.689

The impact of in-room lighting on sleep quality had a mean of 3.94 ± 0.767 indicating that guests find the lighting of average satisfaction in promoting sleep.

Overall, natural lighting in rooms during the day had a mean score of 3.79 ± 0.69 showing average satisfaction, though some rooms might benefit from enhanced natural light.

The mean scores imply that guests generally find the indoor lighting inadequate for mood satisfaction, relaxation and sleep quality highlighting a need for significant improvements in all areas.

The thresholds for interpretation (0.5, 0.6–0.7, etc.) were derived from conventional statistical benchmarks used in descriptive analysis, where standard deviation ranges are used to categorize response variability. These thresholds were aligned with patterns observed during the pilot study, ensuring contextual relevance.

### Proposed guest preference for sustainable lighting strategies

The statistical analysis of sustainable lighting and guest mood satisfaction is given in Table 3. The results indicates that guests had a strong level of mood satisfaction with the concept of sustainable lighting, as reflected by a mean of 3.06 ± 0.84. This indicates that guests are inclined strongly towards the sustainable lighting rather than preferring artificial lights in the resort’s vicinity.

**Table 3:** Sustainable lighting and guest perception.

Sustainable lighting and guest perception	Mean	SD
Familiar with the term “sustainable lighting”	3.06	0.835
Sustainable lighting can contribute to stress reduction	3.94	0.831
Important of hotel or resort uses sustainable lighting practices	4.02	1.078

Despite this, guests generally were found to perceive sustainable lighting as a helpful tool for reducing stress, indicated by a mean score of 3.94 ± 0.83, presenting a promising role in enhancing relaxation, mood & sleep quality. Moreover, guests felt that the importance of resorts adopting sustainable lighting practices to be significant denoted by a mean of 4.02 ± 1.08 which indicates that most guests consider sustainable lighting practices valuable and prefer staying in places that prioritize environmentally responsible lighting ambiances. Overall, the results highlight a general appreciation for sustainable lighting, especially for its psychological benefits.

### Impact of sustainable lighting on psychological performance of guest

The descriptive factors of the model depicting the impact of sustainable lighting on mood is given in Table 4. The observations show that a strong positive relationship exists (R = 0.831), indicating that as sustainable lighting improves, mood tends to improve significantly as well. The R^2^ value of 0.690 suggests that sustainable lighting explains about 69.0% of the variance in mood, meaning it has a substantial influence, though other factors may still contribute to mood.

**Table 4:** Model for mood and sustainable lighting.

R	R square	Adjusted R square	Std. error of the estimate	Change statistics		
				R square change	F change	Sig. f change
0.831	0.690	0.686	0.25312	0.690	218.11	0.001

The t-value for sustainable lighting is 3.403 (shown in Table 5), indicating that the effect of sustainable lighting on mood is statistically significant (p < 0.01). This statistically significant relationship suggests that sustainable lighting plays a practical role in enhancing mood and, therefore, will be an important design consideration in spaces aimed at supporting positive mood, relaxation & sleep quality. This finding also underscores the broader potential of sustainable lighting to foster well-designed spaces that promote both sustainable practices and psychological benefits.

**Table 5:** Coefficient for mood and sustainable lighting.

Model	Unstandardized coefficients		T	Sig
	B	Std. Error		
Constant	2.893	0.241	11.983	0.000
Sustainable lighting	0.219	0.064	3.403	0.001

The influence of sustainable lighting on outdoor environmental lighting experience (guest psychology) shows a weak but statistically significant positive relationship (as reported in Table 6), with R = 0.297. Although this measures only a small effect size, the finding remains significant in terms of design. The R^2^ of 0.088 indicates that sustainable lighting explains 8.8% of the variation in guest well-being, implying other variables also have significant contributions. Yet this low correlation, with statistical significance (p < 0.01), supports that sustainable lighting does make a positive contribution, though not the only one, to increasing psychological comfort in resort environments.

**Table 6:** Model for well-being and Sustainable lighting.

R	R square	Adjusted R square	Std. error of the estimate	Change statistics		
				R square change	F change	Sig. F change
0.297	0.088	0.079	0.32564	0.088	9.367	0.003

The t-value of 3.061 is quite high (shown in Table 7), meaning that the relationship between sustainable lighting and guest psychology is statistically significant (p < 0.01). This means that where more sustainable lighting is implemented, the greater will be the positive impact on well-being and this effect is reliable and repeatable, such that with each unit increase in sustainable lighting, well-being is expected to improve, therefore, it can be confidently stated that sustainable lighting has a positive impact on well-being.

**Table 7:** Coefficient for well-being and Sustainable lighting.

Model	Unstandardized coefficients		t	Sig
	B	Std. error		
Constant	3.279	0.170	19.317	0.000
Sustainable lighting	0.139	0.045	3.061	0.003

## Conclusion

The findings of this study confirm that sustainable lighting significantly enhances guest experiences in coastal beach resorts, particularly in promoting relaxation, comfort, mood enhancement, and sleep quality. Guests consistently valued environments that balanced natural and artificial lighting, incorporated dimmable controls, and used warm-toned LEDs at night. These results align with prior research, such as Aditi & Roy’s work on meditation resorts, which demonstrated the calming effects of warm, daylight-integrated environments, and El-Sayed & Abed’s study showing that sustainable lighting in hotel lobbies fosters positive emotional responses. Heschong’s emphasis on lighting personalization and Zhang et al.’s findings on dynamic LEDs improving circadian regulation further reinforce the link between adaptive lighting and psychological well-being observed in this study.

The unique coastal resort context amplifies these effects. Abundant natural daylight, open layouts, and leisure-oriented guest expectations make daylight integration especially impactful, while the intense sunlight in such environments underscores the need for adaptive artificial lighting to manage glare and thermal comfort. These conditions distinguish coastal resorts from other hospitality typologies, suggesting that location-specific lighting guidelines are necessary to optimize both sustainability outcomes and guest satisfaction.

Beyond immediate guest experience benefits, these findings have strategic relevance for resort ESG (environment, social, governance) performance and green hotel certifications such as LEED and Green Key. Implementing personalized lighting controls, warm-toned LEDs, and daylight-responsive systems supports environmental responsibility while enhancing comfort, meeting both guest expectations and measurable sustainability metrics.

Overall, the study emphasizes the growing importance of sustainable lighting practices, with a significant number of guests valuing environmentally responsible accommodations. These insights suggest that sustainable lighting not only supports energy efficiency but also plays a vital role in enhancing psychological comfort, mood, and overall guest satisfaction in resort environments.

This research contributes to knowledge by offering empirical evidence in the Indian coastal resort context, an area where such data were limited. Given the quantitative evidence of the link between sustainable lighting and psychological performance (mood, relaxation, stress reduction, sleep quality), this study offers a framework for incorporating lighting strategies into hospitality design standards and guest experience models. The findings also reinforce the strategic potential of sustainability, both as an environmental obligation and competitive advantage, in the tourism industry. Future research might build upon this work by considering seasonal variations, cross-cultural guest responses, and long-term impact on guest loyalty to strengthen the case for sustainable lighting as an essential piece of the hospitality design puzzle.

### Recommendations

To enhance guest psychological performance through sustainable lighting in resorts, several key strategies can be implemented, excluding natural light. Providing personalized lighting controls in guest rooms and communal spaces can significantly improve relaxation and comfort. Dimmable lighting and adjustable settings allow guests to tailor the lighting to their preferences, creating a more soothing environment.

The use of warm-toned and energy-efficient LED lighting is also essential in promoting a relaxing atmosphere while ensuring sustainability. Implementing such lighting solutions in guest rooms, dining areas, and social spaces can enhance sleep quality and overall comfort while reducing energy consumption. Thoughtfully designed lighting in dining and communal areas is equally important, as a well-balanced combination of brightness and ambiance fosters a welcoming and enjoyable experience, encouraging positive social interactions.

Prioritizing sustainable lighting solutions further supports environmental goals while enhancing the guest experience. Integrating smart lighting systems that adjust automatically based on time of day or occupancy can optimize energy efficiency without compromising comfort. Additionally, raising awareness about these sustainable practices through informational signage or digital displays in guest rooms and public areas can reinforce eco-friendly initiatives and positively influence guests’ perceptions.

By implementing these strategies, resorts can not only improve guest satisfaction and psychological well-being but also contribute to sustainable tourism by adopting responsible lighting practices.

The survey data strongly supports the conclusion that sustainable lighting practices have a significant impact on guest psychology in beach resorts. The results reveal a clear correlation between the implementation of these strategies and positive outcomes, emphasizing the importance of sustainable lighting in improving the overall guest psychology.

### Ethical approval and consent statement

This study was conducted in accordance with the ethical guidelines of Manipal Academy of Higher Education (MAHE), Manipal. Ethical approval for this research was obtained from the Institutional Ethics Committee (IEC) of Manipal School of Architecture and Planning. Additionally, permission was granted by the respective resorts to conduct case studies for academic purposes.

Prior to participation, all respondents were informed about the purpose of the study, and informed consent was obtained from each participant. Participation was voluntary, and respondents were assured of their anonymity and the confidentiality of their responses.

## Data Availability

"The datasets used and/or analysed during the current study are available from the corresponding author on reasonable request."

## References

[1] Olya, H., Altinay, L., Farmaki, A., Kenebayeva, A. & Gursoy, D. Hotels’ sustainability practices and guests’ familiarity, attitudes and behaviours. *J. Sustain. Tour.* **29**(7), 1063–1081. 10.1080/09669582.2020.1775622 (2021).

[2] Sholanke, A., Fadesere, O. & Elendu, D. The role of artificial lighting in architectural design: A literature review. In *IOP Conference Series: Earth and Environmental Science* (IOP Publishing Ltd, 2021). 10.1088/1755-1315/665/1/012008.

[3] Aryani, S. M., Kusumawanto, A. & Suryabrata, J. A. Lighting in the workplace as the visual environment that affect the occupant’s mood: A literature review. (2020).

[4] Jones, P., Hillier, D. & Comfort, D. Sustainability in the hospitality industry. *Int. J. Contemp. Hosp. Manag.* **28**(1), 36–67. 10.1108/IJCHM-11-2014-0572 (2016).

[5] Emuy, E. V., Alvar, R., Diane, P. C. C. & Gadjamel, I. D. Tourist satisfaction and destination loyalty on beach resorts. *Int. J. Tour. Hotel. Manag.* **6**(1), 08–11. 10.22271/27069583.2024.v6.i1a.80 (2024).

[6] Mehmetoglu, M. & Engen, M. Pine and Gilmore’s concept of experience economy and its dimensions: An empirical examination in tourism. *J. Qual. Assur. Hosp. Tour.* **12**(4), 237–255. 10.1080/1528008X.2011.541847 (2011).

[7] Bakker, I., van der Voordt, T., Vink, P. & de Boon, J. Pleasure, arousal, dominance: Mehrabian and Russell revisited. *Curr. Psychol.* **33**(3), 405–421. 10.1007/s12144-014-9219-4 (2014).

[8] Obeidat, I., Obeidat, S., Rumman, S. A. & Al-Jubouri, F. The role of sustainable interior design and its impact on customer’s behavior in commercial environments. *IOP Conf Ser Earth Environ Sci* **1026**(1), 012054. 10.1088/1755-1315/1026/1/012054 (2022).

[9] Park, N., Pae, J. Y. & Meneely, J. Cultural preferences in hotel guestroom lighting design. *J Inter Des* **36**(1), 21–34. 10.1111/j.1939-1668.2010.01046.x (2010).

[10] Said, D., Youssef, K. & Waheed, H. Energy efficiency opportunities in hotels. *Renew. Energy Sustain. Dev.* **3**(1), 99–103. 10.21622/resd.2017.03.1.099 (2017).

[11] El-Sayed, S. & Abed, M. The use of sustainability principles and lighting technology in lighting hotels’ lobby area. *J. Assoc. Arab. Univ. Tour. Hosp.*10.21608/jaauth.2021.97836.1243 (2021).

[12] Naik, S. & Professor, A. Sustainable coastal tourism development-a geoinformatics approach (2022). [Online]. Available: www.questjournals.org

[13] Juvan, E. & Dolnicar, S. The attitude–behaviour gap in sustainable tourism. *Ann. Tour. Res.* **48**, 76–95. 10.1016/j.annals.2014.05.012 (2014).

[14] Zhang, R. et al. Impacts of dynamic LED lighting on the well-being and experience of office occupants. *Int. J. Environ. Res. Public Health* **17**(19), 7217. 10.3390/ijerph17197217 (2020). 33023141 10.3390/ijerph17197217 PMC7579128

[15] Davis, L. K., Bumgarner, J. R., Nelson, R. J. & Fonken, L. K. Health effects of disrupted circadian rhythms by artificial light at night. *Policy Insights Behav. Brain Sci.* **10**(2), 229–236. 10.1177/23727322231193967 (2023).

[16] Giannoukou, I. Revolutionizing hospitality: Strategic integration of innovation management embracing technological innovation for enhanced customer experiences. *Tech. Bus. Manag.* **7**, 24–39. 10.47577/business.v7i.10585 (2024).

[17] Kaplan, S. The restorative benefits of nature: Toward an integrative framework. *J. Environ. Psychol.* **15**(3), 169–182. 10.1016/0272-4944(95)90001-2 (1995).

[18] Das, A. & Paul, S. K. Artificial illumination during daytime in residential buildings: Factors, energy implications and future predictions. *Appl. Energy* **158**, 65–85. 10.1016/j.apenergy.2015.08.006 (2015).

[19] Jenness, D. The impact of smart room technology on guest experience journal of hotel and business management commentary correspondence to. *J. Hotel Bus. Manag.* **12**, 1000062–1000063. 10.35248/2169-0286.23.12.062 (2023).

[20] Heschong, L. *Visual Delight in Architecture* (Routledge, 2021). 10.4324/9781003097594.

[21] Zhang, Y., Han, M. & Liu, X. Study on demand and intelligence of light environment in guest rooms of high-end resort hotels. [Online]. Available: www.forestchemicalsreview.com

[22] Cajochen, C. et al. Evening exposure to a light-emitting diodes (LED)-backlit computer screen affects circadian physiology and cognitive performance. *J. Appl. Physiol.* **110**(5), 1432–1438. 10.1152/japplphysiol.00165.2011 (2011). 21415172 10.1152/japplphysiol.00165.2011

[23] Bashir, F. M. et al. Effects of natural light on improving the lighting and energy efficiency of buildings: toward low energy consumption and CO2 emission. *Int. J. Low-Carbon Technol.* **19**, 296–305. 10.1093/ijlct/ctad130 (2024).

[24] Bura, A. G. Minimizing light pollution in a forest resort Dr-Parag Govardhan Narkhede BKPS College of Architecture Pune:Government of Maharashtra-India. [Online]. Available: https://www.researchgate.net/publication/360318163

[25] Sophie Lambert. Sustainability in Hospitality | Hotel Lighting. https://occa-design.com/blog/sustainability-hospitality-hotel-lighting/.

[26] Roy, B. C. Role of lighting in built forms with reference to meditation resort. *Int. J. Indian Psychȯl.*10.25215/0803.006 (2020).

[27] Acampora, A., Preziosi, M., Lucchetti, M. C. & Merli, R. The role of hotel environmental communication and guests’ environmental concern in determining guests’ behavioral intentions. *Sustainability (Switzerland)* **14**(18), 11638. 10.3390/su141811638 (2022).

[28] Eloise Boyd. Trend focus: Biophilic hotel and resort design.

[29] Shivanna, M. B. International journal of multidisciplinary educational research tourism development of coastal Karnataka: a geographical study. [Online]. Available: www.ijmer.in

[30] Manjunath, H. & Biradar, S. I. Development of eco-tourism in Karnataka state: An analytical study of jungle lodges and resorts. *EPRA Int. J. Econ. Growth Environ. Issues*10.36713/epra2933 (2019).

